# The efficacy of the dental Water Jet, orthodontic, and conventional toothbrushes in plaque removal around orthodontic braces in adolescents: A randomized controlled trial

**DOI:** 10.1002/cre2.752

**Published:** 2023-07-10

**Authors:** Mhd Hadi Al Hariri, Mawia Karkoutly, Saleh Al Kurdi, Mohammad Alkassar, Nada Bshara

**Affiliations:** ^1^ Department of Pediatric Dentistry Damascus University Damascus Syria

**Keywords:** adolescent, dental plaque, orthodontic brackets, toothbrushing

## Abstract

**Objectives:**

Orthodontic treatment improves both masticatory function and the aspects of facial esthetics through the correct alignment of the teeth. If oral hygiene is neglected during fixed orthodontic treatment, it may lead to plaque accumulation and gingivitis. The aim of this randomized controlled trial was to evaluate the effectiveness of the dental Water Jet (DWJ), and orthodontic toothbrush (O‐TH) in removing dental plaque around the orthodontic braces compared to conventional toothbrush (C‐TH) in adolescents.

**Materials and Methods:**

This was a three‐arm, double‐blind, and parallel‐group randomized active‐controlled trial. Forty‐five patients were randomly allocated into three groups: DWJ, the O‐TH, and the C‐TH (control group). The primary outcome measure was dental plaque accumulation change from the baseline (t_0_) to post‐cleaning (t_1_), and plaque scores were recorded using the Orthodontic Plaque Index (OPI). The current clinical trial was registered and approved by Australian New Zealand Clinical Trials Registry (ACTRN12623000524695).

**Results:**

A statistically significant difference was noted in the OPI scores between different time points in the DWJ group, the O‐TH group, and the C‐TH group (*p* < .05). However, no significant difference was noted between the groups after the cleaning procedure (*p* > .05).

**Conclusion:**

The level of oral hygiene was not satisfactory in patients undergoing fixed orthodontic treatment. In addition, the efficacy of the DWJ was not superior to O‐TH nor to C‐TH in plaque removal.

## 1 INTRODUCTION

Orthodontic treatment improves both masticatory function and the aspects of facial esthetics through the correct alignment of the teeth (Preoteasa et al., 2012). However, some complications of orthodontic treatment might arise with some orthodontic appliances, such as dental caries, tooth discoloration, gingival hyperplasia, and dental plaque accumulation. Some other of the complications arise sometimes but might be prevented (Preoteasa et al., 2012; Wishney, 2017). Moreover, adolescents undergoing orthodontic treatment are more prone to gingival enlargement due to hormonal changes during puberty (Hosadurga et al., 2016). Dental plaque is considered the main factor causing dental caries (Marsh, 2010). Evidence has suggested that oral bacteria cause demineralization in both enamel and dentin. In a hamster model, molars remain intact until they erupt, and dental caries only develop when molars get exposed to oral bacteria (Qiu et al., 2020).

Several changes occur to the oral bacterial flora during fixed orthodontic treatment. Multibracket orthodontic treatment provides additional retentive areas for plaque. In addition, the concentration of acidogenic bacteria such as Streptococcus mutans, and Lactobacillus increased in plaque. Therefore, results in enamel demineralization and the onset of white spots, which is a sign of early decay. White spots form within a month upon the initiation of the orthodontic treatment if oral care is absent. Despite the difficulties in maintaining oral hygiene during orthodontic treatment, it is essential to maintain good oral health to ensure an appropriate quality of life for the patient (Khoroushi & Kachuie, 2017).

It is believed that removing plaque by means of a toothbrush is an effective way to prevent demineralization in patients undergoing orthodontic treatment. However, it is not clear whether using manual toothbrushes alone is sufficient to remove dental plaque (Marçal et al., 2022). Some recommendations called for the use of interdental brushes along with toothbrushing to remove plaque in areas that are “hard to reach” (Rasines, 2009). Several innovations have been proposed and are changing the face of oral care such as the dental Water Jet (DWJ), and the orthodontic toothbrush (O‐TH). The O‐TH is a V‐shaped toothbrush head design that is used due to its efficacy for fixed orthodontic patients with inadequate oral hygiene (Marçal et al., 2022). Evidence has shown that the DWJ has yielded satisfactory outcomes in terms of gingival inflammation and bleeding reduction (Al‐Mubarak et al., 2002; Jolkovsky et al., 1990). In addition, the DWJ is a viable tool for patients with implants or fixed dental prostheses to reduce pathogenic bacteria. Although the efficacy of the DWJ was reported in the literature (Improving, 2019; Jahn, 2010), few studies have evaluated its efficacy in adolescents undergoing fixed orthodontic treatment (Mazzoleni et al., 2019; Sharma et al., 2008). Therefore, the aim of this randomized controlled trial was to evaluate the effectiveness of the DWJ, and O‐TH in removing dental plaque around the orthodontic braces compared with conventional toothbrush (C‐TH) in adolescents. The null hypothesis was that no significant difference would be noted between the DWJ, O‐TH, and C‐TH in removing dental plaque around the orthodontic braces. Such studies provide another dental care tools for maintaining oral hygiene among orthodontic patients.

## 2 MATERIALS AND METHODS

### 2.1 Study design

This was a three‐arm, double‐blind, parallel‐group randomized active‐controlled trial. Ethical approval was provided by the ethics board at Damascus University, and It was conducted according to Helniski Declaration 2013 and to CONSORT statement. This study took place between October 13, 2022, and February 15, 2023, at Damascus University. The current clinical trial was registered and approved by Australian New Zealand Clinical Trials Registry (ACTRN12623000524695).

### 2.2 Recruitment and eligibility criteria

Inclusion criteria

The inclusion criteria were as follows:

1. Healthy children.
2. Children aged 11–15 years.
3. Patients undergoing fixed orthodontic treatment.
4. Patients are during the active phase of orthodontic treatment.
5. Patients with mild crowding teeth.

Exclusion criteria

The exclusion criteria were as follows:

1. Special health care needs patients.
2. Patients with enamel defects.
3. Patients with periodontal or gingival diseases.

Forty‐eight patients from department of orthodontics were assessed for eligibility. An orthodontist standardized the records of patients that are during orthodontic treatment before recruitment. The investigator included healthy patients with no periodontal or gingival diseases according to periodontal screening and recording (PSR) index (Landry & Jean, 2002). Three patients were excluded because they were diagnosed with enamel defects according to enamel defects index (EDI) (Elcock et al., 2006). Hence, 45 patients were included, and written informed consent was obtained from patients’ legal guardians.

### 2.3 Sample size calculation

The sample size was calculated using the G.Power 3.1.9 software (Heinrich‐Hein‐Universität‐Düsseldorf, Germany; http://www.gpow-er.hhu.de/). Effect size *f* = 0.4797282/α err prob = 0.05/Power (1 − *β* err prob) = 0.80/Number of groups = 3. A sample size of 45 patients was obtained and assessed for eligibility. A sample size of 45 patients was obtained and randomly allocated into three groups according to the method used in dental plaque removal around the orthodontic braces. Each group included 15 patients.

### 2.4 Randomization

Study participants were randomly allocated into three groups in a ratio 1:1:1 using simple randomization method. The same number of participants were randomly assigned to each treatment group. The random allocation sequence was performed using randomization online software; https://www.randomizer.org. The number of sets was 3 with 15 patients per sets, and the number range was from 1 to 45.

### 2.5 Blinding

This was a double‐blind, randomized controlled trial. Both the independent data collector and the statistician were blinded but blinding the study participants was not possible. The data collector was kept unaware of which intervention arms patients have been assigned to and the statistician is unaware of which records belong to which arm.

### 2.6 Procedures

The primary outcome measure was dental plaque accumulation change from the baseline (*t*_0_) to postcleaning (*t*_1_) using DWJ, O‐TH, or C‐TH, and plaque scores were recorded using the Orthodontic Plaque Index (OPI). In addition, OPI assessed the inflammatory condition of the adjacent marginal gingivae. Dental plaque was disclosed using disclosing solution (Mira‐2‐Ton®, Hager & Werken GmbH & Co. KG) after 4 h of the last brushing then immediately after cleansing. The dentition is divided into sextants, and the highest score of plaque accumulation per sextant was assigned. The OPI for a patient was determined by calculating the average. The plaque accumulation around the bracket vicinity was screened as follows:

1. Bracket vicinity is plaque‐free.
2. Plaque deposits on one tooth surface at the bracket vicinity.
3. Plaque deposits on two tooth surfaces at the bracket vicinity.
4. Plaque deposits on three tooth surfaces at the bracket vicinity.
5. Plaque deposits on four tooth surfaces at the bracket vicinity and/or an indicator for gingivitis.

Group 1. The DWJ: Participants were instructed on how to use the DWJ flosser with the orthodontic jet tip (Water Jet, JollyDent) before the dental cleaning procedures. The flosser tip was set at 90° angle to the tooth surface and for 3 s per tooth. The DWJ was set at medium pressure (70 psi) and 1200 pulses per minutes. The overall cleansing time was around 2 min.

**Figure 1:**
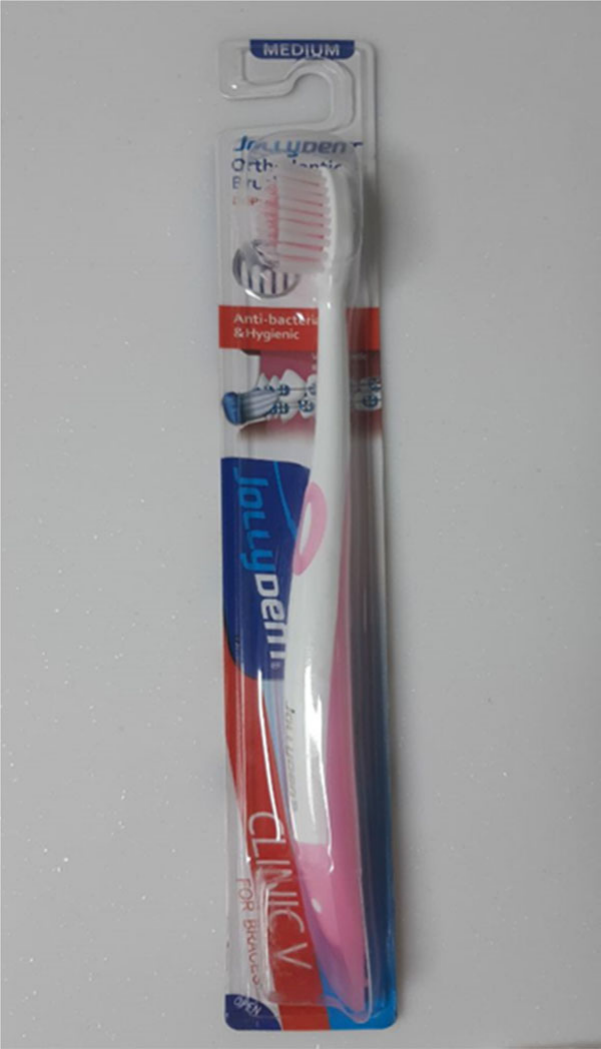
Orthodontic toothbrush.

Group 2. The O‐TH: Participants were instructed on how to use the modified Bass technique, using V‐shaped bristle O‐TH (Clinic V, JolleyDent) (Figure 1), and antiplaque toothpaste (Elgydium, UniPharma Co., Ltd).

**Figure 2:**
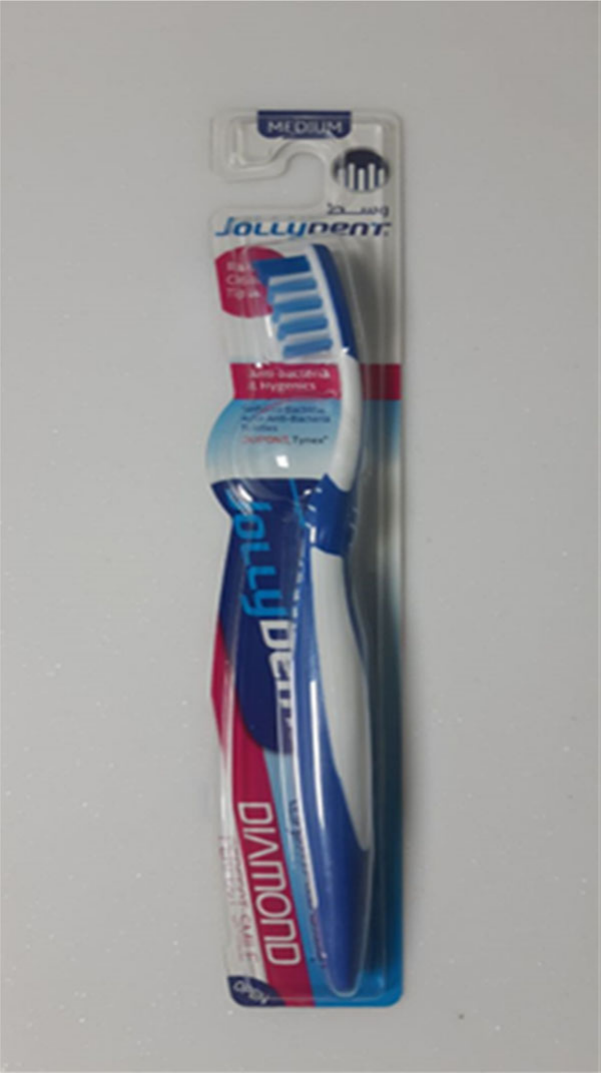
Conventional toothbrush.

Group 3. The C‐TH (control group): Participants were instructed on how to use the modified Bass technique, using flat trim bristle C‐TH (JolleyDent) (Figure 2), and antiplaque toothpaste (Elgydium, UniPharma Co., Ltd).

No additional oral hygiene measures were taken in the study groups.

### 2.7 Statistical analysis

IBM SPSS software version 24 (IBM Corp.) was used for statistical analysis. Graphs were created using excel (Microsoft Excel, Microsoft Corp). Nonparametric tests were applied since that data was not normally distributed. Kruskal–Wallis and Wilcoxon signed‐rank tests were used to compare non‐paired and paired data, respectively. The level of Significance was set at *p* < .05.

## 3 RESULTS

**Figure 3:**
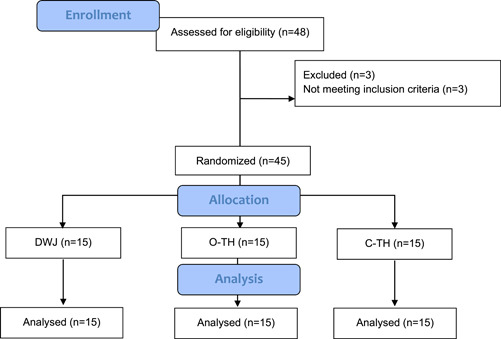
CONSORT flow diagram.

The CONSORT flow diagram is presented in Figure 3. A total of 48 participants were assessed for eligibility, and 45 were randomized. More than half of the participants were female (*n* = 27; 60%). The mean age was 12.8 years (standard deviation [SD] 1.37; range 11–15 years). OPI scores were presented as mean, SD, maximum, and minimum for each group at baseline and post‐cleaning (Table 1).

**Table 1:** Descriptive statistics of the OPI scores at the baseline and after cleaning procedure.

Group	*n*	Time point	Mean ± SD	Minimum	Maximum
DWJ	15	*t*_0_	3.13 ± 0.91	2.62	3.64
		*t*_1_	1.66 ± 0.72	1.26	2.06
O‐TH	15	*t*_0_	2.86 ± 0.51	2.58	3.15
		*t*_1_	1.4 ± 0.63	1.04	1.75
C‐TH	15	*t*_0_	3.4 ± 0.5	3.11	3.68
		*t*_1_	1.4 ± 0.5	1.11	1.68

Abbreviations: C‐TH, conventional toothbrush; DWJ, the dental Water Jet; OPI, Orthodontic Plaque Index; O‐TH, orthodontic toothbrush; SD, standard deviation; *t*_0_, baseline; *t*_1_, postcleaning.

**Figure 4:**
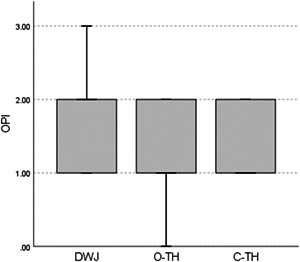
Box plots of the postcleaning OPI scores showing median, interquartile range, minimum, and maximum. C‐TH conventional toothbrush; DWJ, the dental Water Jet; OPI, Orthodontic Plaque Index; O‐TH, orthodontic toothbrush.

**Figure 5:**
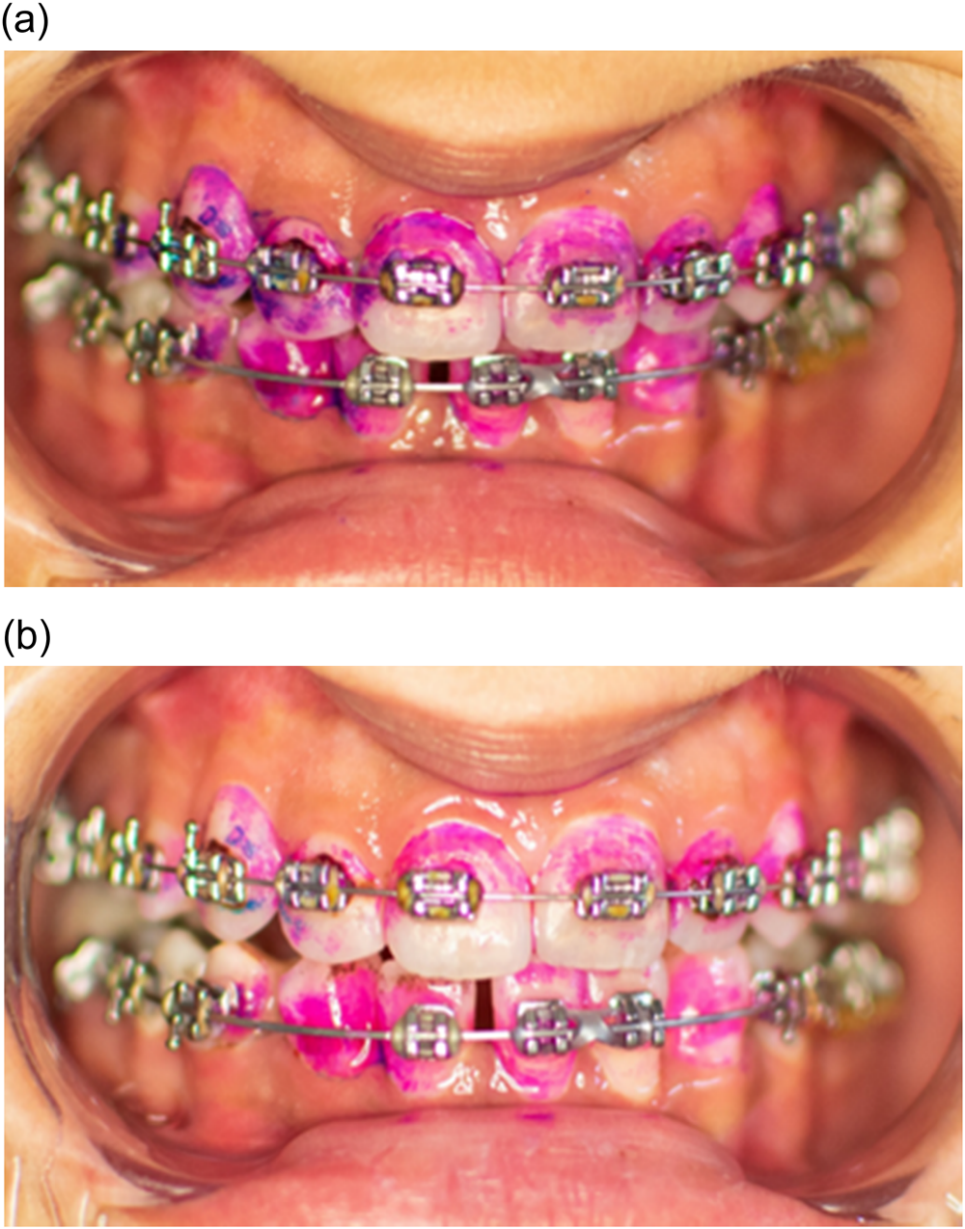
Photographs showing the dental plaque accumulation. (a) At baseline. (b) postcleaning with DWJ. DWJ, the dental Water Jet.

**Figure 6:**
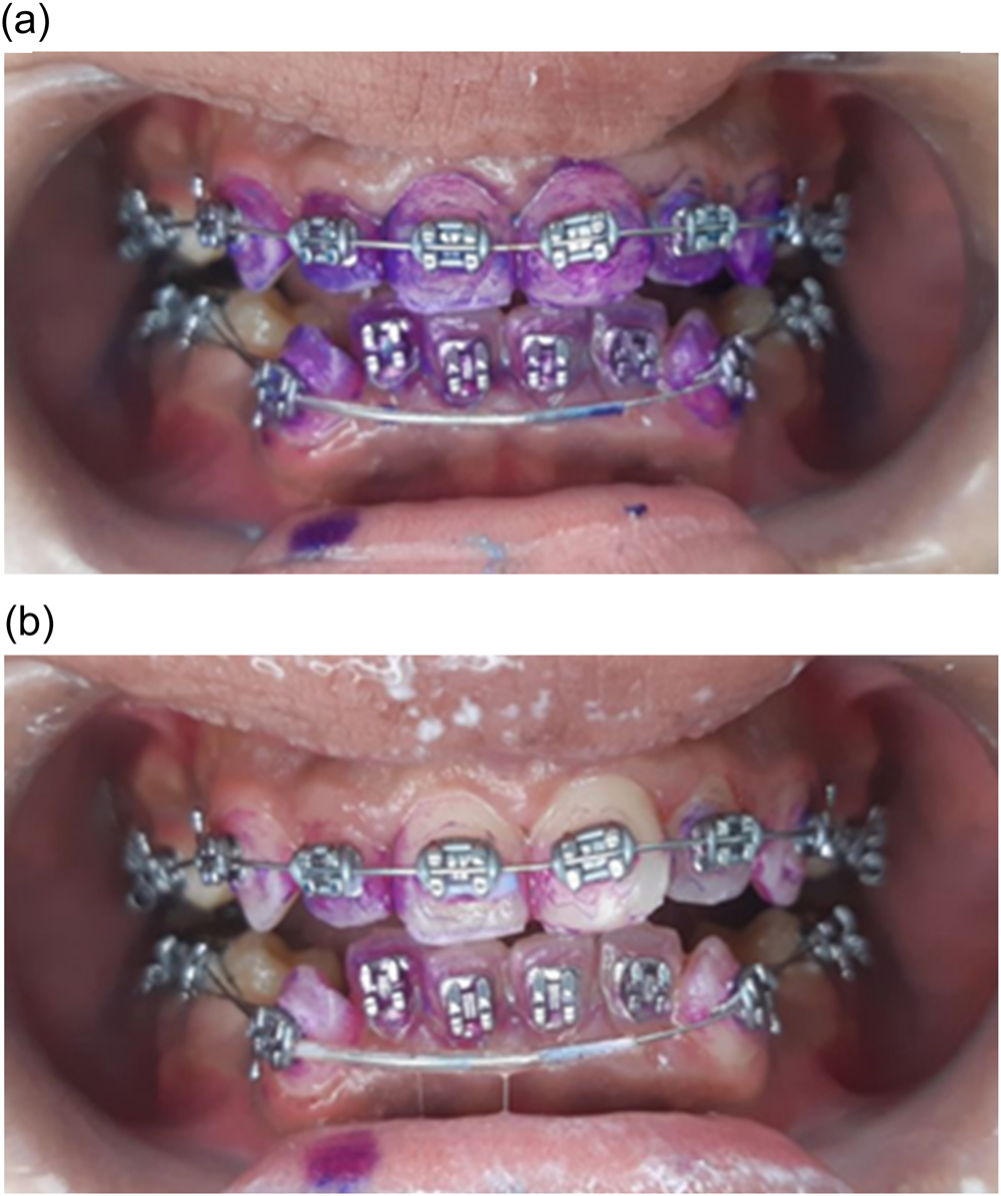
Photographs showing the dental plaque accumulation. (a) At baseline. (b) postcleaning with the C‐TH. C‐TH conventional toothbrush.

**Figure 7:**
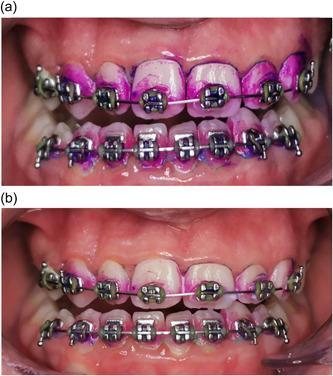
Photographs showing the dental plaque accumulation. (a) At baseline. (b) postcleaning with the O‐TH. O‐TH, orthodontic toothbrush.

No significant difference was observed between the OPI scores at the baseline *p* = (0.104), suggesting that the study groups were well‐balanced and homogeneous in terms of plaque accumulation (Table 2). In addition, no significant difference was noted between the groups *p* = (0.326) after the cleaning procedure (Table 3 and Figure 4). Multiple comparisons were not performed, because the overall test did not show significant differences across the samples. However, a statistically significant difference was noted in OPI scores between different time points in the DWJ group *p* = (0.001) (Figure 5), the O‐TH group *p* = (0.000) (Figure 6), and the C‐TH group *p* = (0.000) (Figure 7), and (Table 4).

**Table 2:** Comparison results of Kruskal–Wallis test of the OPI scores between groups at baseline.

Group	*N*	*df*	*p* Value
DWJ	15	2	.104
O‐TH	15		
C‐TH	15		

Abbreviations: C‐TH conventional toothbrush; *df*, degrees of freedom; DWJ, the dental Water Jet; OPI, Orthodontic Plaque Index; O‐TH, orthodontic toothbrush.

**Table 3:** Comparison results of Kruskal–Wallis test the OPI scores between groups postcleaning.

Group	*N*	*df*	*p* Value
DWJ	15	2	.567
O‐TH	15		
C‐TH	15		

Abbreviations: C‐TH conventional toothbrush; *df*, degrees of freedom; DWJ, the dental Water Jet; OPI, Orthodontic Plaque Index; O‐TH, orthodontic toothbrush.

**Table 4:** Comparison results of Wilcoxon signed‐rank test dental plaque accumulation between the two‐time points.

Groups		Ranks average	Ranks total	Test value	*p*‐Value
DWJ	**Negative Ranks**	7.5	105	−3.372	.001*
	**Positive Ranks**	0	0		
O‐TH	**Negative Ranks**	8	120	−3.508	.000*
	**Positive Ranks**	0	0		
C‐TH	**Negative Ranks**	8	120	−3.499	.000*
	**Positive Ranks**	0	0		

Abbreviations: C‐TH conventional toothbrush; DWJ, the dental Water Jet; O‐TH, orthodontic toothbrush.

* *p* < .05 = significant difference.

## 4 DISCUSSION

This study was conducted due to the high demand for additional preventive methods to effectively reduce the incidence of dental caries around orthodontic braces (Wishney, 2017). The C‐TH is one of the most common methods of controlling plaque for patients undergoing fixed orthodontic treatment. However, there are several methods that outperform the efficacy of the C‐TH (Gomez‐Pereira et al., 2022). Several innovations have been proposed and are changing the face of oral care, such as the DWJ, and the O‐TH. However, to date, the plaque removal efficacy of the O‐TH has not yet been fully validated (Rasines, 2009). To the best of our knowledge, this is the first randomized controlled trial that compares the efficacy of the DWJ, the O‐TH, and the C‐TH in removing dental plaque around orthodontic braces in adolescents.

Orthodontic treatment is a risk factor for dental caries, due to the accumulation of plaque around the orthodontic braces. If oral hygiene is not maintained during treatment, this leads to plaque buildup and gingivitis (Khoroushi & Kachuie, 2017). Therefore, controlling dental plaque is the cornerstone of caries prevention and proper oral health (Marsh, 2010). According to Kumar et al. (2021) and Mazzoleni et al. (2019), there is an increase in gingival inflammation, recession, and plaque accumulation after fixed orthodontic treatment. A possible explanation for this is that plaque‐retentive areas are increased due to the fixed orthodontics appliances.

In this current study, the OPI was used to screen plaque accumulation around the bracket vicinity due to its concise, and quick documentation (Beberhold et al., 2012). Plaque disclosing agent was used in its solution form because it is more user‐friendly for fixed orthodontic patients.

The current study used the modified bass brushing technique, it is relatively easy to learn, effective, and widespread. In addition, it provides better gingival and interproximal cleaning, and it does not induce gingival recession (Poyato‐Ferrera et al., 2003). An O‐TH V‐shaped toothbrush head design was used due to its efficacy for fixed orthodontic patients with inadequate oral hygiene (Schätzle et al., 2017a).

In this study, the DWJ was set at a medium pressure of 70 PSI because it yielded satisfactory outcomes in terms of plaque removal and preserving enamel thickness and microhardness (Aziz & Gonzalez, 2022). The orthodontic jet tip was used because Gorur et al. (2009) confirmed that the use of this tip removes 99.84% of dental plaque compared with the standard jet tip.

No statistically significant difference was noted between the DWJ and the C‐TH in the efficacy of plaque removal. This result is in agreement with Mazzoleni et al. (2019) findings, suggesting that the DWJ was not beneficial for home oral hygiene in patients with multi‐bracket fixed appliances. In addition, Al Husseini et al. (2008) suggested that the oral irrigator was not effective in reducing plaque when used as an adjunct to C‐TH. Moreover, Gallie et al. (2019) found that the oral irrigator was not superior to the C‐TH alone at 3 months. On the contrary, Sharma et al. (2008) found that DWJ was effective in plaque removal and gingival bleeding control. In addition, according to Hurst and Madonia (1970), the adjunct use of the oral irrigator with conventional toothbrush was more effective in reducing aerobic flora, and lactobacillus count than toothbrushing alone. Hence, it is recommended to use several supportive means rather than imposing a single cleaning method to improve oral health (Richards, 2018). This variation in results could be attributed to the different study designs. In addition, the encouraging results of earlier studies could be explained by the “novelty effect”, which means that people tend to perform better when they encounter a new technology or innovation (Shin et al., 2019).

In the current study, the O‐TH was not superior to C‐TH in plaque removal, this result is consistent with Rafe et al. (2006) and Habar and Timo (2022) findings. In addition, Gomes et al. (2012), stated that the selection of toothbrush should be based on comfort since O‐TH did not outperform C‐TH in plaque removal. However, Schätzle et al. (2017b) suggested that the O‐TH has a superior plaque removal efficacy for teeth with orthodontic braces. This variation could be attributed to the fact that in vitro studies are likely to occur in a more controlled environment.

In the current study, no statistically significant difference was found between the DWJ and the O‐TH. This result agrees with the one reported by Soni et al. (2012), which suggested that both O‐TH and DWJ are effective oral hygiene tools.

A statistically significant difference was noted in dental plaque accumulation before and after cleaning in both groups. These results are in agreement with Gomes et al. (2012) and Kurnianti and Razi (2019) findings. This could be explained by the fact that the participants were instructed on how to remove dental plaque using different means. Hence, patients need more training in removing dental plaque, whether by traditional or supportive methods. Furthermore, their level of oral hygiene was not satisfactory.

The current study has strengths. First, the study groups were well‐balanced and homogeneous in terms of plaque accumulation at the baseline. Secondly, Both the data collector and the statistician were blinded. However, the main limitation of this study was the inability to blind the study participants.

## 5 CONCLUSIONS

Within the limitations of this study, the level of oral hygiene was not satisfactory in patients undergoing fixed orthodontic treatment. In addition, the efficacy of the DWJ was not superior to O‐TH nor to C‐TH in plaque removal. Thus, one can use the most suitable toothbrush for them.

## AUTHOR CONTRIBUTIONS

Mhd Hadi Al Hariri collected data; Mawia Karkoutly wrote the manuscript; Saleh Al Kurdi extracted the data and performed the statistical analysis; Mohammad Alkassar collected data; and Nada Bshara performed critical revision of the manuscript. All authors have read and approved the manuscript.

## CONFLICT OF INTEREST STATEMENT

The authors declare that there are no conflicts of interest.

## ETHICS STATEMENT

Ethical approval was provided by the ethics board at Damascus University.

## Data Availability

The data sets generated during and/or analyzed during the current study are available from the corresponding author on reasonable request.
